# Dissecting the Membrane Association Mechanism of Aerolysin Pores at Femtomolar Concentrations Using Water as a Probe

**DOI:** 10.1021/acs.nanolett.4c00035

**Published:** 2024-10-29

**Authors:** Tereza Roesel, Chan Cao, Juan F. Bada Juarez, Matteo Dal Peraro, Sylvie Roke

**Affiliations:** † Laboratory for Fundamental BioPhotonics (LBP), Institute of Bioengineering (IBI), and Institute of Materials Science (IMX), School of Engineering (STI), and Lausanne Centre for Ultrafast Science (LACUS), École Polytechnique Fédérale de Lausanne (EPFL), CH-1015 Lausanne, Switzerland; ‡ Department of Inorganic and Analytical Chemistry, School of Chemistry and Biochemistry, University of Geneva, 1211 Geneva, Switzerland; § Institute of Bioengineering, School of Life Sciences, École Polytechnique Fédérale de Lausanne, CH-1015 Lausanne, Switzerland

**Keywords:** aerolysin, nanopores, angle-resolved second harmonic scattering, molecular sensing, sequencing

## Abstract

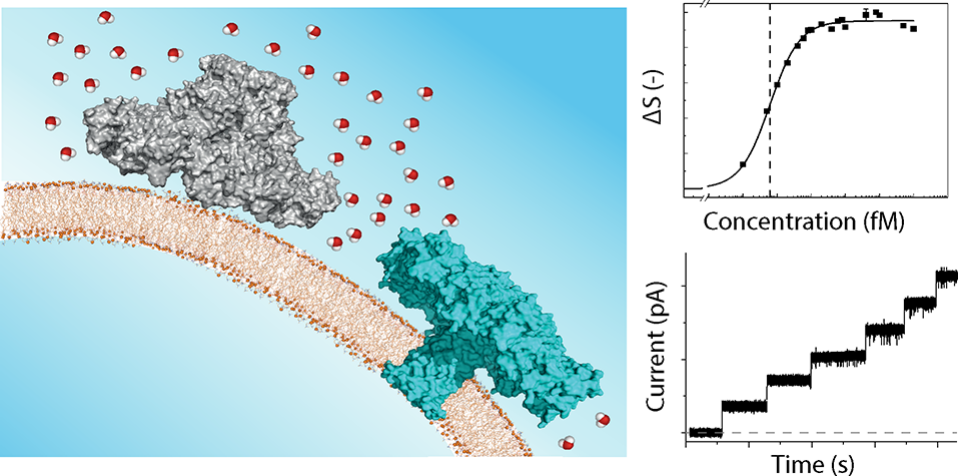

Aerolysin is a bacterial toxin that forms transmembrane pores at the host plasma membrane and has a narrow internal diameter and great stability. These assets make it a highly promising nanopore for detecting biopolymers such as nucleic acids and peptides. Although much is known about aerolysin from a microbiological and structural perspective, its membrane association and pore-formation mechanism are not yet fully understood. Here, we used angle-resolved second harmonic scattering (AR-SHS) and single-channel current measurements to investigate how wild-type (wt) aerolysin and its mutants interact with liposomes in aqueous solutions at femtomolar concentrations. Our AR-SHS experiments were sensitive enough to detect changes in the electrostatic properties of membrane-bound aerolysin, which were induced by variations in pH levels. We reported for the first time the membrane binding affinity of aerolysin at different stages of the pore formation mechanism: while wt aerolysin has a binding affinity as high as 20 fM, the quasi-pore and the prepore states show gradually decreasing membrane affinities, incomplete insertion, and a pore opening signature. Moreover, we quantitatively characterized the membrane affinity of mutants relevant for applications to nanopore sensing. Our study provides a label-free method for efficiently screening biological pores suitable for conducting molecular sensing and sequencing measurements as well as for probing pore-forming processes.

Pore-forming proteins are a class of proteins targeting the plasma membrane of a variety of organisms and are usually involved in defense or attack mechanisms.^1^ The most extensively characterized pore-forming proteins are the bacterial pore-forming toxins (PFTs), which are classified as helical (α-) or beta-sheet (β)-PFTs depending on the secondary structure of their transmembrane region.^1,2^ Most commonly, pore-forming proteins are expressed as soluble proteins that subsequently oligomerize upon protease activation and/or membrane receptor binding, converting to a transmembrane pore.

Aerolysin, a β-PFT produced by *Aeromonas sp.*, is the founding member of a large superfamily that spans all of the kingdoms of life.^1,3,4^ Aerolysin is expressed as an inactive precursor, proaerolysin, which contains 4 distinct domains: domain 1 (in gray, Figure 1A) is involved in binding N-linked oligosaccharides while domain 2 (in blue) is a glycosyl phosphatidylinositol (GPI)-anchored binding region, domain 3 (in yellow, called stem loop) is responsible for the oligomerization process, and domain 4 (in green) contains a C-terminal peptide (CTP, in red) that is required for folding into the soluble monomeric form.^5^ Proteolysis of the CTP allows aerolysin to oligomerize in a heptameric ring-like complex that inserts into the target membrane to form the pore after passing through several distinct intermediate structures (namely prepore, post pre-pore, and quasi-pore intermediates; Figure 1B–D).^6,7^ These stages are defined by a different length and completion of the β-barrel that is formed after the stem loop undergoes a conformational change upon oligomerization. First, a soluble transient pre-pore state is formed, which was captured *in vitro* only by the stabilizing Y221G mutation; this state is characterized by two concentric β-barrels, held together by hydrophobic interactions^4^ (Figure 1B). Next, in the series of events putatively leading to pore formation, the inner β-barrel fully extends toward the membrane passing from a quasi-pore state captured by the stabilizing mutation K246C-E258C (Figure 1C) and ending in the mature aerolysin pore (Figure 1D). The structure of each of these states is known at high resolution from a combination of molecular modeling and cryo-EM experiments,^4,8^ which has led to a crude understanding of the sequential steps leading to pore formation.

**Figure 1:**
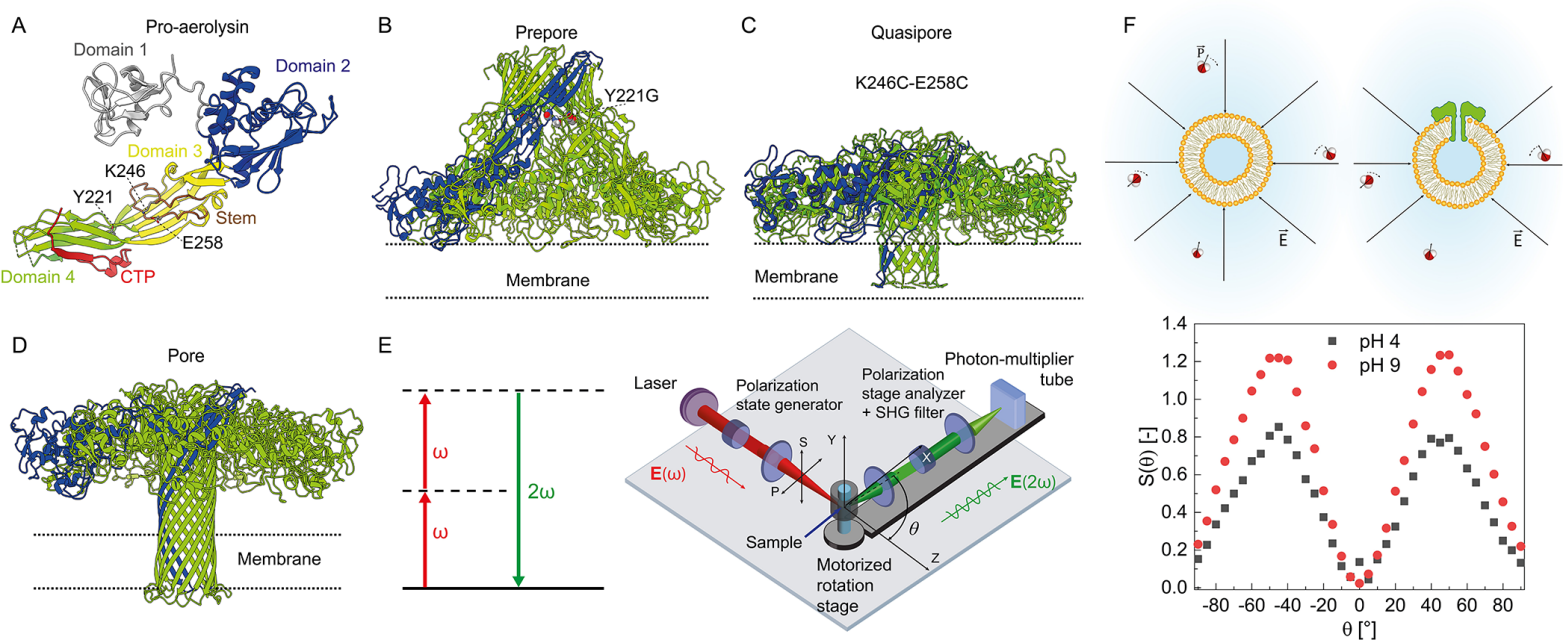
Structure of aerolysin in different states and how to measure its membrane association. **A.** Structure of monomeric pro-aerolysin (PDB: 1PRE): Domain 1 in gray, domain 2 in blue, domain 3 in yellow, and domain 4 in green. Illustration of the aerolysin structure in prepore (**B**), quasi-pore (**C**), and pore (**D**) states and labels of the various mutations with localization in panel **A**. The images were generated in UCSF ChimeraX.^9^**E.** Energy-level scheme and sketch of the AR-SHS experiment. P(S) refers to the polarization state of the beam parallel (perpendicular) to the scattering plane. All measurements were recorded with all beams polarized in the horizontal plane. For the single-angle experiments, the scattering angle θ was set to 45°, corresponding to the angle with maximum scattering intensity. Adapted with permission from ref (20). Copyright [2024] [American Physical Society] **F.** Top: Illustration of how interfacial electric fields (***E⃗***) orient dipolar water (indicated by the symbol ***p⃗***) and how this is affected by aerolysin–membrane interaction. Here, charge-water interaction is the most relevant physical mechanism for creating the SH contrast. Bottom: Example SHS pattern of LUVs composed of 99:1 mol % DOPC:DOPA membranes interacting with 5 × 10^–10^ M wt aerolysin in aqueous solutions having pH values of 4 (black data) and 9 (red data). One mol % DOPA was used to increase the signal-to-noise ratio for these measurements. The maximum interfacial SH intensity occurs around θ = 45°. Figure S1 shows how this graph was generated.

Recently, aerolysin has received much attention, not only because of its biological role, but also because of its potential use in biotechnological applications.^10,11^ Due to its stability, easy incorporation into lipid bilayers, and a narrow internal diameter of the pore lumen (∼1 nm), aerolysin is nowadays one of the most promising biological nanopores, permitting the detection of several biopolymers such as nucleic acids, peptides, and oligosaccharides.^12−16^ We and others have shown that point mutations within the pore can be performed for specific molecular sensing, thereby achieving enhanced sensitivity and selectivity.^10,17,18^ At the same time, we observed that the mutations have an impact on the pore formation efficiency. This is, in fact, crucial since without efficient incorporation of the pore into the lipid bilayer and a strong lipid–pore interaction, nanopore experiments cannot be reliably conducted. Therefore, to explore new biological or *de novo* designed nanopore candidates, it would be highly desirable if one could estimate lipid–protein interactions at very low concentrations and without sacrificing the sample. Such a technique would be a useful tool to screen for pore mutants that have stable and strong lipid interactions and would provide insights into their membrane association mechanism. It would be instrumental in identifying promising nanopore candidates for molecular sensing experiments.

To better understand the complex aerolysin–membrane interplay, we use a recently introduced method called high-throughput angle-resolved second harmonic scattering (AR-SHS).^19^ In this method, two fs pulsed near-infrared photons with frequency ω interact with an aqueous solution containing liposomes. A nonresonant second-order polarization is created in only those regions of the sample where there is an anisotropic distribution of anisotropic molecules. This polarization is composed of displaced charge density that oscillates at twice the frequency of the incoming light (2ω), and it emits photons at this second harmonic frequency, which are detected. The AR-SHS experiment is illustrated in Figure 1E. It was recently shown that for liposome–water and other membrane systems, interfacial water molecules can be used as a contrast agent.^20−24^ Thus, this technique is a label-free interface-specific method with molecular sensitivity toward the orientational distribution of interfacial water. Recent studies have shown that changes in the membrane water can help understand temperature-induced phase transitions^22^ and map different protein–membrane interactions, such as the interaction of α-synuclein with the aqueous environment of liposomes.^23^ It has also been shown that AR-SHS enables the retrieval of the protein–membrane binding constant, which was exemplified by the interaction of perfringolysin O (PFO) with liposomes.^25^ These measurements demonstrate the ability of AR-SHS to ultrasensitively detect protein–liposome interactions in an aqueous solution in the fM–pM range, corresponding to a single transmembrane protein bound to a single liposome. This permits the use of small volumes of a protein solution.

Here, we used this approach to quantify the interaction between aerolysin pore-forming proteins and lipid membranes (Figure 1). Our results clearly show that AR-SHS can capture pH-dependent changes in the surface charge of the aerolysin cap region. Combining the ultrasensitive AR-SHS measurements with single-channel current recording experiments, we quantitatively analyzed the binding affinities of different aerolysin mutants to the lipid membrane. We estimated a binding affinity of 20 fM for wt aerolysin at pH 7.4. Furthermore, we extracted the dissociation constants of two mutants—the first one mutated in the cap region (i.e., pore entry), R220A, and the second one mutated in the stem region (i.e., pore exit), K238N. We observed binding affinities varying by 2 orders of magnitude between these two mutants, with *K*_*d*_ going from 10^–14^ M to 10^–12^ M for K238N and R220A, respectively. Additionally, we compared the binding behavior of mutation Y221G (Figure 1B) and of mutation K246C-E258C (Figure 1C), and no complete pore opening was observed for either of these mutants. However, the quasi-pore exhibited binding behavior similar to that of wt aerolysin, but with a binding affinity more than an order of magnitude smaller.

## Surface Charge Detection upon pH Changing Conditions

**Figure 2:**
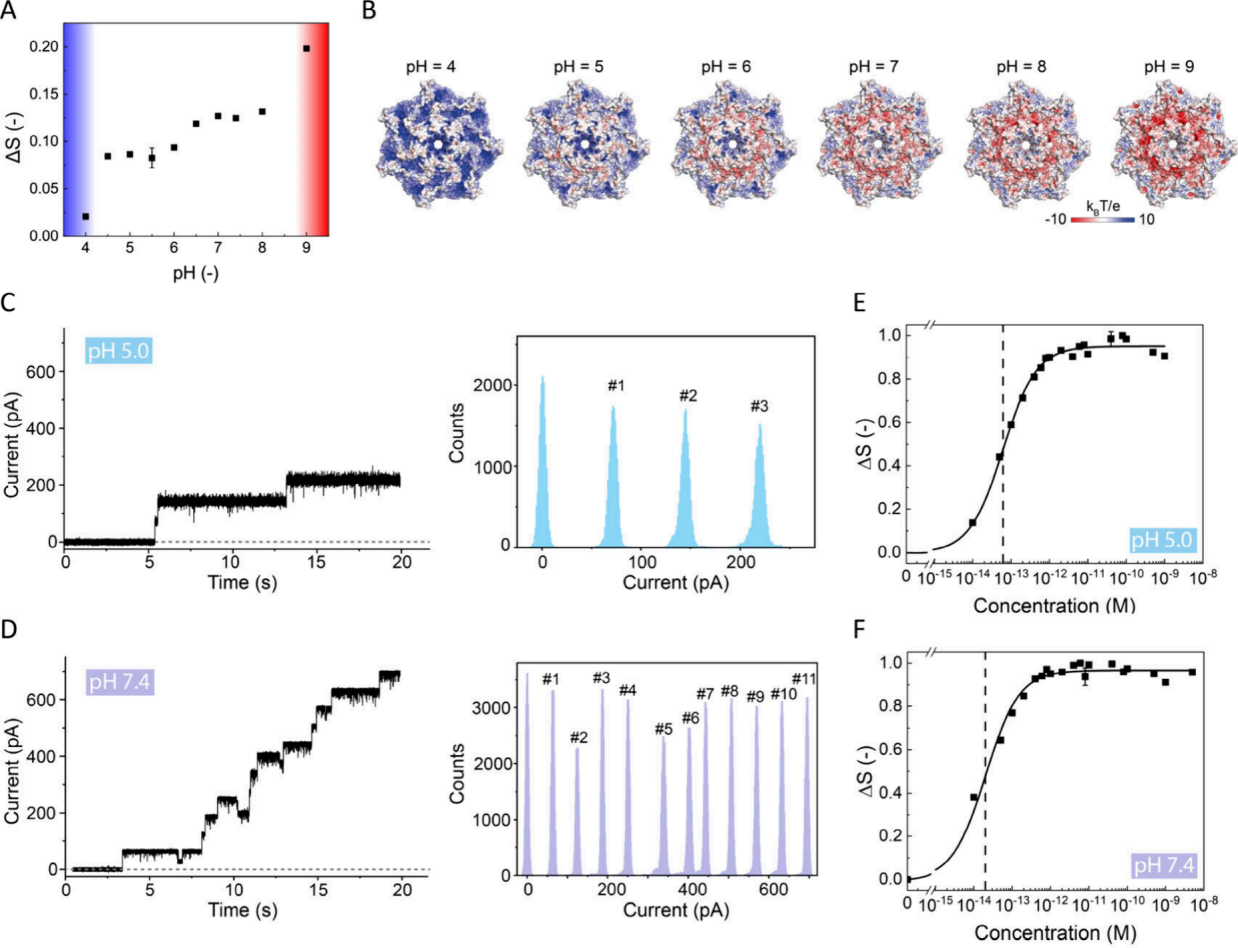
pH-dependent surface charge and membrane binding affinities. **A.** Second harmonic intensity difference relative to bulk water (S) at the scattering angle with maximum intensity (*θ*_*max*_ = 45°) vs pH of the aqueous solution. The measurements were performed with DOPC liposomes and wt aerolysin. The blue and red highlighted areas indicate the positive and negative surface charges on the cap region of aerolysin. **B.** Electrostatic potential mapped on the cross sections of the aerolysin cap domain obtained via the APBS Web server^26^ depicting the charge distribution of the pore (blue: positive charge; red: negative charge) visualized in PyMol.^27^**C, D.** A representative single-channel current recording traces of the aerolysin-DOPC free-standing membrane system in aqueous solution at pH 5 (**C**) and 7.4 (**D**), with the corresponding histogram current distribution, where each step indicating that another pore incorporation is counted (numbered by #). **E, F.** Normalized SH intensity difference (*ΔS*) at the angle with the maximum intensity (*θ*_*max*_ = 45°) vs wt aerolysin concentration on the logarithmic scale at pH 5 (**E**) and 7.4 (**F**). The data are fitted using eq S2, giving a dissociation constant of *K*_*d*_ = (6.2 ± 0.4) × 10^–14^ M for pH 5 and *K*_*d*_ = (2.0 ± 0.2) 10^–14^ M for pH 7.4 (represented by a dashed line). The error bars were determined as the standard deviation from 100 measurements for all of the AR-SHS measurements. The AR-SHS measurements were performed with liposomes composed of 99:1 mol % DOPC:DOPS liposomes interacting with wt aerolysin.

Figure 2A shows the recorded change (*ΔS*) in the normalized SH intensity as a function of the pH. This plot was created by recording PPP-polarized SH intensities at the angle of maximum intensity (*θ*_*max*_ = 45°, Figure 1F and Figure S1) of liposome solutions containing 0.5 nM wt aerolysin and of identical solutions that contained no liposomes. Normalized SHS intensities were then computed via eq S1 and compared to liposome solutions containing no aerolysin, resulting in *ΔS* values, which were measured as a function of pH. *ΔS* is defined as the absolute difference between the normalized SH intensity of liposomes with a given concentration of aerolysin in the solution and liposomes without added aerolysin (eq S2, Materials and Methods)

The interaction of charge with dipolar water molecules results in orientational directionality (Figure 1F top). Water molecules facing a positively charged surface will tend to orient with the O atoms toward the surface. This mechanism can be used to determine the electrostatic surface potential.^20^ As such, the SH response is very sensitive to the amino acid charges of the protein. Note that the DOPC membrane is, on average, charge neutral. At pH 4, the normalized SH intensity (eq S1, Materials and Methods) is the lowest at *ΔS* = 0.02. At pH 4.5, the normalized SH intensity sharply increases by 0.06, after which it smoothly increases until pH 8, at which point it rapidly increases to 0.2. The electrostatic potential on the surface of the extracellular part of the wt aerolysin over pH change is computed with the help of the APBS Web server^26^ (see Method F in the SI) and reported in Figure 2B. This result shows how the protonation state of the amino acids present in the cap domain significantly changes with pH, producing a mostly positively charged pore at pH 4 and a negatively charged one at pH 9. It is interesting that significant variations in the normalized intensity occur in correspondence with the p*K*_a_ values of the relevant titratable residues (i.e., Asp/Glu, His and Arg/Lys) within the pH range explored (Table S1 shows the p*K*_a_ values). When the pH is lower than 4, Asp and Glu residues are mostly protonated, which reduces the negative charge of the pore cap domain (Figure 2B). On increasing the pH, the net charge of the cap domain becomes more negative with significant steps at pH 6 (deprotonation of His) and pH 9 (deprotonation of Arg and Lys).

The trend observed in Figure 2A can be explained by considering the changes in the orientation of the water molecules that interact with aerolysin. It was previously shown that DOPC liposomes in an aqueous solution have water molecules weakly but preferentially oriented with their H atoms toward the membrane surface.^20^ At pH 4, the inserted aerolysin is positively charged, which forces some water molecules to orient their O atoms toward the aerolysin surface, balancing out the effect of the water orientation induced by the DOPC membrane and resulting in a minimal SH intensity. Between pH 4.5 and 8, the aerolysin cap domain is mostly neutral or slightly negative. In this pH range, the orientation of water is primarily determined by water–DOPC interactions, resulting in a weak preference for H atoms to face the surface. Above pH 8, the aerolysin becomes more negatively charged, and more water molecules reorient with their H atoms toward the surface, causing an increase in the SH intensity. Therefore, the AR-SHS response is a very sensitive marker of the protonation state of the amino acids of aerolysin. We expect this to be a general trend for all transmembrane proteins possessing titratable moieties under variable pH conditions.

## Membrane Binding Affinity of wt Aerolysin Pores

Subsequently, the binding of aerolysin to the membrane was probed using AR-SHS and single-channel current measurements at pH 5.0 and 7.4. In single-channel current recording experiments (Figure 2C and D), the incorporation of an aerolysin pore into a DOPC free-standing membrane was followed by a rapid increase in current when a voltage was applied. This current is proportional to the number of pores present in the membrane. For the aerolysin–DOPC–membrane system in an aqueous solution at pH 5.0, three aerolysin pores were observed in a 20 s recording time, whereas at pH 7.4, many more pores were incorporated into the bilayer under the same conditions and equal recording time. Figure 2E and F shows the coherent SH intensity difference *ΔS* as a function of the wt aerolysin concentration in the solution. *ΔS* increased rapidly between 10 and 100 fM aerolysin, after which it stayed constant. This increase occurred at lower concentrations at pH 7.4 compared to pH 5.0. The leveling off represents the saturation of the interactions, where no additional proteins could insert into the membrane. Using eq S2, we obtained wt aerolysin dissociation constants of *K*_*d*_ = (6.2 ± 0.4) × 10^–14^ M and *K*_*d*_ = (2.0 ± 0.2) × 10^–14^ M for pH 5 and pH 7.4, respectively. The single-channel current and AR-SHS measurements together demonstrate that wt aerolysin has a slightly lower membrane affinity under acidic conditions compared to neutral pH, and this is reflected by less efficient incorporation into lipid bilayers (Table S2).

## Membrane Binding Affinities of Aerolysin Mutants

Having the ability to detect protonation changes of the amino acids of wt aerolysin, we aimed to detect the changes caused by mutating amino acids, particularly those in the cap or the stem region. We used two single point mutations defined by distinctive steric hindrance and electrostatics.^10^ By replacing R220 in the cap region with alanine (R220A), it was shown that the pore diameter becomes two times wider while the insertion efficiency is lowered.^10^ A mutation at the constriction point in the stem region, where K238 is replaced with an asparagine (K238N), was shown instead to prolong the dwell time for DNA sensing with respect to the wt pore.^10^

**Figure 3:**
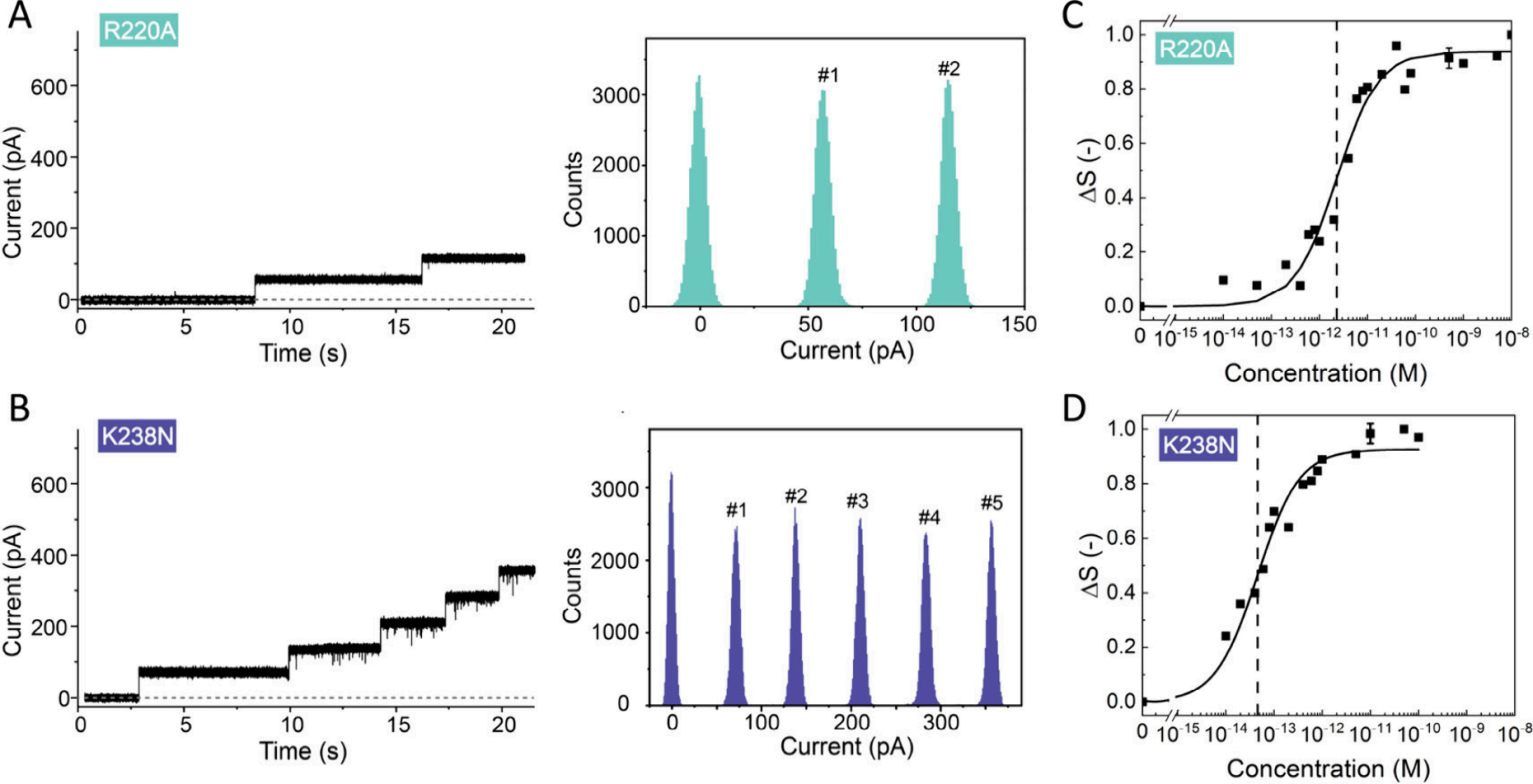
Aerolysin single-point mutant-lipid membrane binding in aqueous solution. **A,B.** A representative single-channel current recording measurement of R220A (**A**) and K238N (**B**) measured with a DOPC free-standing membrane at pH 7.4. **C, D.** SH intensity difference (*ΔS*) at the angle with maximum intensity (*θ*_*max*_ = 45°) vs R220A (**C**) and K238N (**D**) concentration on the logarithmic scale at pH 7.4. The data are fitted using eq S2, giving dissociation constants of *K*_*d*_ = (2.3 ± 0.3) 10^–12^ M and *K*_*d*_ = (4.6 ± 0.6) 10^–14^ M for R220A and K238N, respectively (the dashed line represents the dissociation constant). The data in parts **C** and **D** were measured with DOPC doped with 1% DOPS liposomes. The error bars were determined as the standard deviation from 100 measurements for all of the AR-SHS measurements. The dotted line represents the fitted *K*_*d*_ value for each aerolysin we tested in **A** and **B**.

We investigated the insertion of these mutants in comparison with the wt aerolysin at pH 7.4, employing single-channel current recording and AR-SHS measurements (Figure 3). From the single-channel current recordings at pH 7.4, we observed that two R220A pores and five K238N pores are formed on the membrane (Figure 3A and B). Using the same pores, we performed AR-SHS measurements to obtain the dissociation constant and compared it to that of wt aerolysin. We observed that in the case of R220A, the increase was more gradual than for the wt, starting at less than 1 pM in the mutant concentration and increasing to 100 pM, where it leveled off (Figure 3C). In the case of K238N, the SH intensity difference increased in a similar concentration range (10 fM–1 pM), but more gradually than in the case of wt. It saturated at around 1 pM, without a further change in SH intensity (Figure 3D). Using eq S2, we obtained dissociation constants of *K*_*d*_ = (2.3 ± 0.3) × 10^–12^ M and *K*_*d*_ = (4.6 ± 0.6) × 10^–14^ M for R220A and K238N, respectively. The extracted dissociation constant of K238N at pH 7.4 is slightly higher than that of the wt aerolysin, revealing that K238N has a slightly less favorable interaction with the membrane, confirmed by less incorporation into the membrane. The R220A dissociation constant is 2 orders of magnitude larger than that of wt aerolysin, reflecting the notion that it is less effective at creating pores in the membrane, a finding that is confirmed by the current measurements (Figure 3A, Table S2). Comparing the number of aerolysin pore incorporations in the free-standing bilayer with the dissociation constants obtained from AR-SHS shows that the two techniques return a consistent and quantitative description of the membrane association process.

## Dissecting Membrane Association throughout the Pore-Forming Process

**Figure 4:**
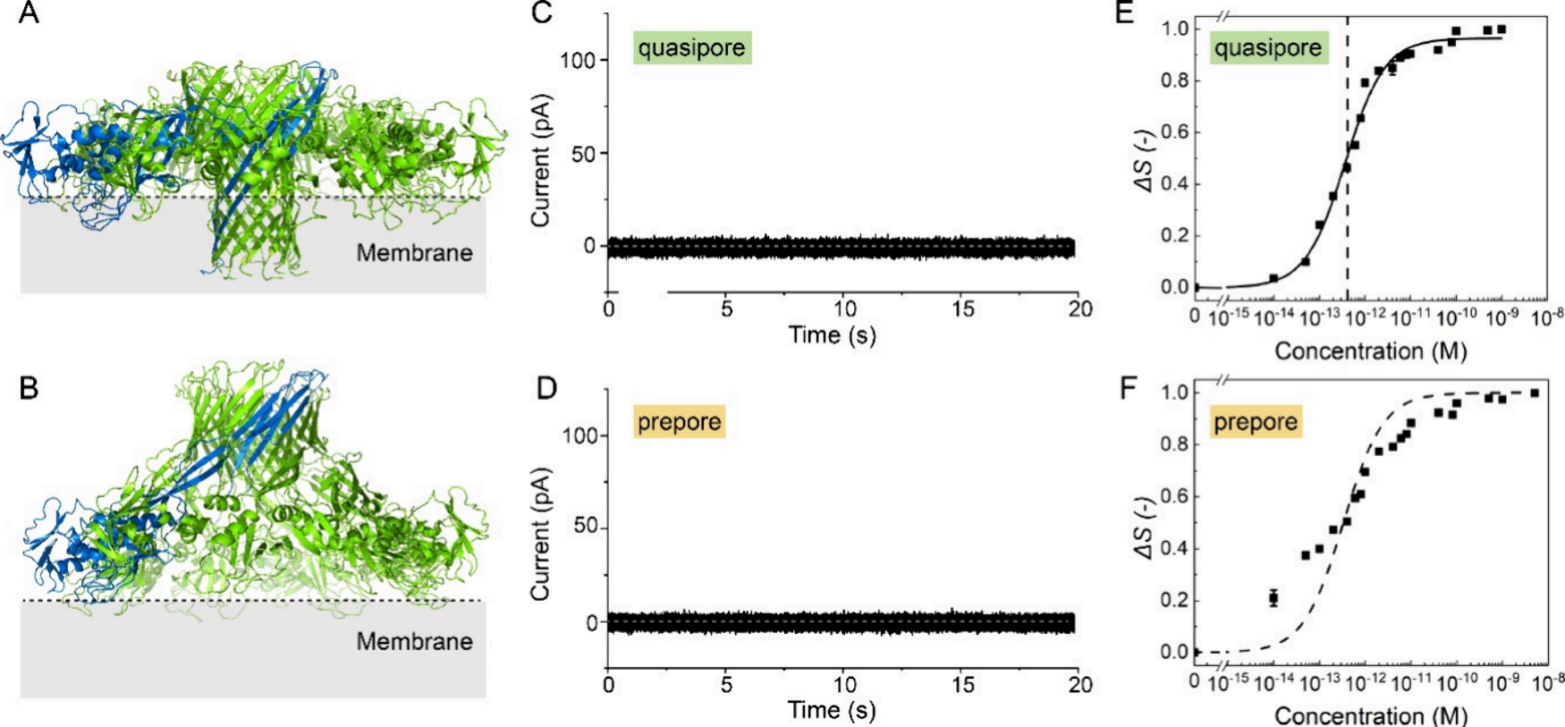
Membrane properties of aerolysin mutants blocked at different stages of pore formation. **A,B.** Structural model of the aerolysin quasi-pore (**A**) and prepore (**B**). The images were generated in UCSF ChimeraX.^9^**C, D.** Representative single-channel current recording measurements of the quasi-pore (**C**) and prepore (**D**) at pH 7.4 on a DOPC free-standing membrane. **E, F.** SH intensity difference (*ΔS*) recorded at the scattering angle having the maximum intensity (*θ*_*max*_ = 45°) vs K246C-E258C (**E**) and Y221G (**F**) concentration on a logarithmic scale. The data were fitted using eq S2, giving a dissociation constant of *K*_*d*_ = (4.1 ± 0.3) × 10^–13^ M for K246C-E258C (quasi-pore). These data were measured with DOPC doped with 1% DOPS liposomes at pH 7.4. The error bars were determined as a standard deviation from 100 measurements for all of the AR-SHS measurements. The dotted line represents the fitted *K*_*d*_ value for each aerolysin we tested in **A**.

To dissect more directly the mechanism of pore formation, we investigated how different pore intermediates interact with the membrane (Figure 4A and B). The Y221G mutant is known to form a prepore state in which the prestem loop is prevented from moving away from the five-stranded β-sheet in the same domain and thereby blocks the hemolytic activity of the toxin^8,28^ while still being able to transiently interact with the target membrane even though it is fully hydrophilic.^28^ The K246-E258C mutations block the pore formation in a later stage of the formation of full transmembrane β-barrel due to the creation of a disulfide bridge between mutated residues 246 and 258.^5^

Figure 4C and D shows zero current during the entire time of the single-channel current measurement, thereby proving that there are no (or very few) pores inserted into the membrane (Table S2). The SH intensity difference (*ΔS*) as a function of the concentration of aerolysin mutants at pH 7.4 is depicted in Figure 4E and F. For the quasi-pore, *ΔS* increased from 10 fM to 10 pM, at which point it saturated. The extracted dissociation constant for the quasi-pore is *K*_*d*_ = (4.1 ± 0.3) × 10^–13^ M, thus more than 1 order of magnitude higher than for wt aerolysin (Figure 4E). The molecular interpretation of these results is that quasi-pores can bind efficiently to the membrane because they have an almost complete β-barrel that can insert into the membrane, but they cannot fully pierce the membrane and are thereby unable to form conducting pores, leading to a decreased binding affinity with respect to the wt pore. The combination of these results implies that the K246C-E258C mutant inserts into the membrane but is unable to transition into a mature pore state, a finding suggested previously.^28^ In the case of Y221G, *ΔS* increased but did not follow the same trend (Figure 4F). The fitting procedure indeed failed for this data set, as indicated by the dashed line in Figure 4F. This could mean that the mutant is transiently interacting with the membrane but not fully anchored. The absence of detectable current in the single-channel recordings agrees with this assessment and is consistent with the fact that the Y221G prepore mutation is known to form fully soluble pores and the barrel region is only partially folded, with its hydrophobic stem region still being protected (Figure 4B).

In summary, by using single-channel current experiments on free-standing membranes and AR-SHS measurements on aerolysin–LUV interactions in aqueous solutions, we have shown that different mutants of aerolysin have distinct types of interaction with the membrane, leading to various degrees of pore formation and targeted insertion. Thanks to the high interfacial and charge sensitivity of AR-SHS, we were able to observe changes in the surface charge of the cap region of aerolysin bound to the LUV for different pH conditions. The charge of the cap region changes from positive at pH 4 to highly negative at pH 9, which is consistent with the predicted electrostatic properties of the pore. This combination of techniques provides a unique method to assess the electrostatic properties of a pore’s surface, which is of special interest for nanopore applications since the changes in the surface can induce a strong electroosmotic flow, enabling the capture of all kinds of biomolecules regardless of their charges.^29^

By combining AR-SHS and single-channel current measurements, we compared the binding affinities of wt aerolysin at different pH values. We extracted the dissociation constants *K*_*d*_ = (6.2 ± 0.4) × 10^–14^ M and *K*_*d*_ = (2.0 ± 0.2) × 10^–14^ M for pH 5.0 and 7.4, respectively. These are 2 orders of magnitude higher than for the interaction of PFO with a cholesterol-rich lipid membrane.^25^ Additionally, we studied the interactions of aerolysin mutants, R220A and K238N, and observed a difference of around 2 orders of magnitude in the *K*_*d*_ of these two mutants, going from 10^–14^ to 10^–12^ M for K238N and R220A, respectively. Lastly, we examined the different binding behavior of aerolysin mutants at different stages of pore formation. No open pore was observed for either quasi-pore or prepore mutants in single-channel current measurements, but the binding of the quasi-pore was measured and demonstrated the extreme sensitivity of the AR-SHS technique, which could be measured at as low as 10 fM.

The combination of using low volumes (10 μL) and low protein concentrations and with the possibility of varying the temperature, pH, or membrane composition means that the AR-SHS method can distinguish which aerolysin pores are efficiently incorporated into the lipid bilayer. Thereby, it provides the opportunity to dissect the molecular features of membrane association along the pore formation pathway or use it for the screening of pore variants with enhanced membrane incorporation and sensitivity. Furthermore, as shown here, AR-SHS alone can successfully characterize (membrane) protein interactions in a label-free environment, and its combination with single-channel current recordings provides a promising method for bionanotechnology.

## Supporting Information Available

Additional experimental details and materials and methods for protein expression, single-channel recordings and AR-SHS experiments including Figure S1 showing the polar plots of raw data and resultant S(θ) values, Table S1 containing the number of amino acids in aerolysin, and Table S2 containing the number of experiments performed for each mutant (PDF)
