# Supplementary material for: Dissecting the Membrane Association Mechanism of Aerolysin Pores at Femtomolar Concentrations Using Water as a Probe
Source: Nano Lett. 2024 Oct 29;24(44):13888–94. doi: 10.1021/acs.nanolett.4c00035 (PMC11544699; doi:10.1021/acs.nanolett.4c00035)
Supplement: Supplementary file 1 — nl4c00035_si_001.pdf [file nl4c00035_si_001.pdf]

Supporting Information for

Dissecting the Membrane Association Mechanism of Aerolysin Pores at Femtomolar Concentrations Using Water as a Probe

Tereza Roesel[1,#], Chan Cao[2,#], Juan F. Bada Juarez[3], Matteo Dal Peraro[3,*], Sylvie Roke[1,*]

[1]*Laboratory for fundamental BioPhotonics (LBP), Institute of Bioengineering (IBI), and Institute of Materials Science (IMX), School of Engineering (STI), and Lausanne Centre for Ultrafast Science (LACUS), École Polytechnique Fédérale de Lausanne (EPFL), CH-1015, Lausanne, Switzerland*
[2]*Department of Inorganic and Analytical Chemistry, School of Chemistry and Biochemistry, University of Geneva, 1211 Geneva, Switzerland*
[3]*Institute of Bioengineering, School of Life Sciences, École Polytechnique Fédérale de Lausanne*

[#] *These authors contribute equally*

[*]*Corresponding authors: MDP (matteo.dalperaro@epfl.ch), SR (sylvie.roke@epfl.ch)*

MATERIALS AND METHODS

A. Chemicals

Lipids 1,2-dioleoyl-*sn*-glycero-3-phosphocholine (DOPC), and 1,2-dioleoyl-*sn*-glycero-3-phospho-L-serine (sodium salt) (DOPS), were purchased in powder form (>99%) from Avanti Polar Lipids (Alabama, USA) and stored at −20 °C until further use. Chloroform for spectroscopy Uvasol® (≥99%, Merck), methanol (≥99.9%, Fisher Chemical), sodium acetate (≥99%, Sigma-Aldrich), acetic acid (≥99.7%, Sigma-Aldrich), sodium phosphate dibasic (≥99%, Fluka), sodium phosphate monobasic (≥99%, Fluka), Trizma hydrochloride (≥99%, Sigma-Aldrich), Trizma base (≥99.9%, Sigma-Aldrich), MES hydrate (≥99.5%, Merck) and sodium chloride (≥99.999%, Acros) were used as received. Deconex 11 UNIVERSAL (Borer Chemie) was used as a cleaning solution. Water was purified by a Milli-Q UF-Plus instrument from Millipore, Inc., and it has an electrical resistivity of 18.2 MΩ·cm. All glassware was washed with a 5% deconex cleaning detergent solution in the ultrasonic bath for 30 min, then they were cleaned with Milli-Q ultrapure water in the sonication bath for another 20 min. After the cleaning, the glassware was rinsed with ultrapure water.

B. Pro-aerolysin expression and purification

Pro-aerolysin wt and mutants were expressed using a pET22b vector, which allows periplasmic expression of the toxin and with a hexa-His tag on the C-terminus, as previously described[1,2]. Mutagenesis was carried out by using the QuickChange II XL kit (Agilent Technologies). Briefly, BL21(DE3)pLysS *E. Coli* containing the wt or the mutant aerolysin expression plasmid were grown at 37°C up to an OD_{600} of 0.6-0.7. Isopropyl β-D-1-

thiogalactopyranoside (IPTG) at a final concentration of 0.25 mM was added, and the temperature was decreased to 20°C for protein production overnight. Cells were harvested, when the OD_{600} reached 1.2, and resuspended in 20 mM sodium phosphate, 500 mM NaCl, pH 7.4 supplemented with cOmplete Protease Inhibitor cocktail (Roche). The cells were lysed by sonication and the supernatant was centrifuged at 12,000*rpm* for 35 min at 4°C, and loaded onto a HisTrap chelating column (GE Healthcare) running on an AKTA™ FPLC workstation. The protein was eluted in a 20 mM sodium phosphate buffer pH 7.4, 0.5 M NaCl buffer with a linear gradient of imidazole (0–0.5 M). Finally, fractions containing the protein were buffer-exchanged in 20 mM Tris, 500 mM NaCl, pH 7.4 by using a HiPrep Desalting column (GE Healthcare) before snap freezing and storing at −80°C.

C. Sample preparation

Large unilamellar vesicles (LUVs) were prepared by the lipid film hydration method followed by extrusion, as described earlier[3,4]. Lipid solutions were created by dissolving 10 mg of lipid powder in chloroform in a round-bottom glass tube. To evaporate the chloroform a gentle stream of N_2 was directed into the rotating glass tube. The residual chloroform was dried under a room temperature vacuum for at least 3 h. The lipid film that was deposited on the glass wall was hydrated in 1 mL of ultrapure water that was heated up to above the phase-transition temperature of the respective lipids that were used. The resulting multilamellar vesicle solutions were extruded through a 100 nm diameter polycarbonate membrane in a Mini extruder (Avanti Polar Lipids). The LUVs were prepared in 50 μM NaCl solution. LUVs were stored in closed containers for up to a week at 4 °C. The size of the resulting large unilamellar vesicles (LUVs) was determined by dynamic light scattering (DLS) using a ZetaSizer Nano ZS (Malvern Instruments Ltd., UK). The z-average diameter of the vesicles in the sample is a result of three averaged measurements. The diameters of the DOPC LUVs were 118 nm with a PDI of 0.07 and their ζ-potential values were -2 ± 8 mV. The DOPC doped with 1% DOPS LUVs were 126 nm in diameter with a PDI of 0.04 and their ζ-potential values were -36 ± 16 mV. Once we added $5x10^{-10}$ M of wt aerolysin, R220A, Y221G, or K246C-E258C the DOPC doped with 1% DOPS LUVs were 127 nm with a PDI of 0.05, 129 nm with a PDI of 0.06, 127 nm with a PDI of 0.05, and 127 nm with a PDI of 0.06, respectively. The concentration of total lipids was 0.5 mg/mL for SHS, DLS, and electrokinetic measurements. The ionic strength of the buffers and aerolysin stock solutions was kept the same for all the SHS, DLS, and electrophoretic measurements. The aqueous solution consisted of 50 μM NaCl, 13.3 μM MES buffer and 100 μM of buffer for pH adjustment. For pH between 4 and 5 Na acetate buffer was used, in the range from pH 6 to 7 phosphate buffer was used, and for the pH from 7.4 and 9 we used Trizma buffer. The pH of the solution was measured using a pH meter (HI 5522

pH/ISE/EC bench meter and HI 1330 pH electrode). The measured pH was always within less than 0.16 points from the desired value. The sample volume used for SHS was 800 µL. Purified wt and mutants of aerolysin were activated as previously described[2]. Briefly, the toxin was diluted to the concentration of 0.2 µg/ml and then incubated at 4°C with Trypsin-agarose (Sigma-Aldrich Chemie GmbH, Buchs, SG Switzerland) for 2 hours to activate the toxin for oligomerization. The solution was centrifuged (10,000*g*, 4°C, 10min) to remove the trypsin-agarose beads. Activated toxins were aliquoted and kept at -80°C. Protein concentration was checked by Nanodrop and added accordingly.

D. Second-harmonic scattering instrument

Figure 1E shows a sketch of the AR-SHS setup, previously described in Ref 5. AR-SHS measurements were performed using 190-fs laser pulses centered at 1028 nm with a 200 kHz repetition rate. The polarization of input pulses was controlled by a Glan-Taylor polarizer (GT10-B, Thorlabs) in combination with a zero-order half-wave plate (WPH05M-1030). The filtered (FEL0750, Thorlabs) input pulses with a pulse energy of 0.3 µJ (incident laser power P = 60 mW) were focused into a cylindrical glass sample cell (inner diameter 4.2 mm) with a beam waist of $2\omega_0 \approx 36$ µm and a corresponding Rayleigh length of ~0.94 mm. The scattered SH beam generated was analyzed (GT10-A, Thorlabs), filtered with a notch filter (ZET514/10x, Chroma), collimated with a plano-convex lens (f = 5 cm), and finally focused into a gated photomultiplier tube (H7421-40; Hamamatsu). The data points were acquired as an average of 100 measurements with a 1.5 s integration time and a gate width of 10 ns. The detection angle θ, which has an acceptance angle of 11.4°, was set to 45°, which corresponds to the angle of the maximum SH intensity. The normalized SH intensity is the SH intensity of the sample from which we subtract the SH intensity of the bulk solution, and this value is normalized by the incoherent SH contribution of bulk water (more details are provided hereafter, see Eq. S1). The SHS patterns for pH 4 and pH 9 are shown in Figure 1F (bottom panel). All measurements were performed in a temperature- and humidity-controlled room (T = 297 K; relative humidity, 26.0 %).

Data interpretation and example scattering patterns

In a non-resonant SHS experiment, a pulsed femtosecond near-infrared laser beam interacts with a liposome solution. SH photons are emitted from all non-centrosymmetric molecules that are non-centrosymmetrically distributed in the sample. Since interfacial water is non-centrosymmetrically distributed while the bulk liquid is not, SHS has an exquisite interfacial sensitivity. When LUVs are dispersed in the water the orientation of water molecules will be perturbed at the interface of these objects. The interfacial water outnumbers lipids with a ratio of 1:>100. The non-resonant nature of the process results in emission of electromagnetic fields

at 2ω with comparable magnitude. The amplitude of this field is proportional to the number of molecules that emit it. Since the coherent SH intensity depends quadratically on the emitted electric field, the effective intensity ratio for lipid:water scales with 1:>10^4, making the AR-SHS method effectively an interfacial water probe. Thus, the interfacial SHS intensity generally reports on membrane hydration arising from the net orientational order of water molecules along the surface normal.

The normalized SH intensity at the angle was calculated as

$$S(\theta) = \frac{I^{PPP}_{sample}(\theta) - I^{PPP}_{solvent+aerolysin}(\theta)}{I^{SSS}_{H_2O}(\theta)}, \quad \text{(S1)}$$

where $I^{PPP}_{sample}(\theta)$ and $I^{PPP}_{solvent+aerolysin}(\theta)$ are the average SHS intensities of the sample (solvent, liposomes, and aerolysin) and solvent with aerolysin, respectively. PPP and SSS represent the polarization state of the outgoing and incident light relative to the scattering plane (P - parallel or S - perpendicular). This eliminates the response from the bulk solution and normalizes the difference relative to water, such that the value is comparable to any previous measurement that was performed in the same manner. It also eliminates possible variations due to the use of different focusing optics or slight variations in alignment. Figure S1 shows an example of how $S(\theta)$ is generated. It shows a polar plot of the recorded raw data for computing the response plotted in Fig. 1F for pH. Fig. S1A shows the raw data (intensity) recorded as a function of scattering angle, plotted as a polar plot. The arrow indicates the direction of the incoming near IR beam, and the scattering data is recorded in the horizontal plane with all beams polarized in the same direction. The SH scattering pattern of the LUVs composed of 99:1 mol% DOPC:DOPA membranes interacting with 5 x 10^{-10} M wt aerolysin in aqueous solution (black data) has a double lobed appearance, while the scattering pattern of the buffer solution containing 5 x 10^{-10} M wt aerolysin in aqueous solution (blue data) has a single lobe. The SSS SH scattering pattern of ultrapure water (red data) is angle independent. Fig. S1B shows the resulting AR-SHS pattern (green data) retrieved from Eq. S1 as a function of scattering angle, plotted as a polar plot. This AR-SHS pattern reports only on changes in the orientational distribution of water that arise from the interface[4].

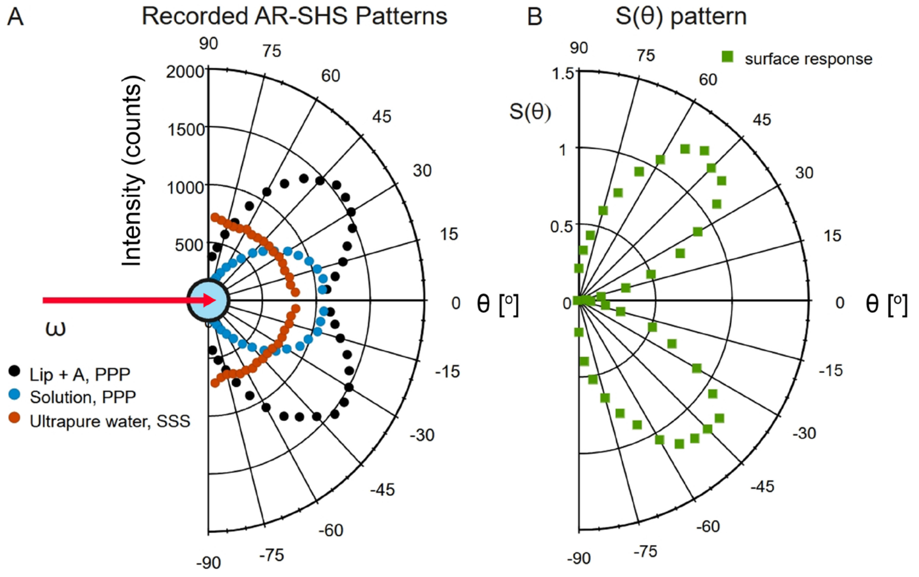

Figure S1: Polar plots of raw data and resultant S(θ) values. A: The PPP SH scattering pattern of the LUVs composed of 99:1 mol% DOPC:DOPA membranes interacting with 5 x 10^{-10} M wt aerolysin in aqueous solution (black data), the buffer solution containing 5 x 10^{-10} M wt aerolysin in aqueous solution (blue data), and the SSS SH scattering pattern of ultrapure water (red data). B: The resulting PPP AR-SHS pattern (green data) retrieved from Eq. S1 as a function of scattering angle.

The normalized coherent SH intensity difference ΔS was calculated for each concentration of aerolysin as a difference between normalized SH intensity of LUVs without added aerolysin $S_L(\theta)$ and LUVs with the given concentration of aerolysin, $S_{LP}(\theta)$, $\Delta S = |S_L - S_{LP}|$. This value was then normalized to 1 for better comparison between the samples. The fitting procedure was previously described in more detail in Ref. 6. The measured difference in the coherent SH intensity ΔS is

$$\Delta S = \frac{c}{K_d + c}, \quad \text{(S2)}$$

where c is the aerolysin concentration and K_d is the dissociation constant. The error bars were determined as a standard deviation from 100 measurements. As a result of the aerolysin concentration increase (adding volume), the particles in the solution were getting diluted so the linear correction for the number of particles in the focal spot was applied. In the dissociation constant calculations, we assumed each protein insertion was a unique event.

E. Single-channel current recording experiments

Phospholipid of 1,2-dioleoyl-*sn*-glycero-3-phosphocholine powder (Avanti Polar Lipids Inc., Alabaster, AL, USA) was dissolved in octane (Sigma-Aldrich Chemie GmbH, Buchs, Switzerland) to a final concentration of 8 mg/mL. Purified wt and mutants of aerolysin were activated as previously described[2]. Briefly, the toxin was diluted to the concentration of 0.2 µg/ml and then incubated at 4°C with Trypsin-agarose (Sigma-Aldrich Chemie GmbH, Buchs, SG Switzerland) for 2 hours to activate the toxin for oligomerization. The solution was centrifuged (10,000*g*, 4°C, 10min) to remove the trypsin-agarose beads. Activated toxins were aliquoted and kept at -80°C. Nanopore single-channel current recording experiments were performed on an Orbit Mini instrument equipped with a temperature control setup (Nanion, Munich, Germany). Phospholipid membranes were formed across a MECA 4 recording chip that contains 4 circular microcavities (size 50 µm diameter) in a highly inert polymer. Each cavity contains an individual integrated Ag/AgCl microelectrode and can record four artificial lipid bilayers in parallel. The buffers for the different experiments include 10 mM phosphate buffer pH 7.4, 10 mM Tris pH 9, and 10 mM sodium acetate pH 5 all complemented with 1M KCl. The temperature was set to 25°C for all experiments. Once a stable lipid baseline with a capacitance also above 6 pF, the activated toxins were added and insertion(s) of the pore was recorded for each aerolysin mutants (40 nM final concentration) and recorded with Elements Data Reader (Elements srl, Italy) and further analyzed by using Clampfit (Axon, Molecular device). Experiments were repeated at least 3 times to obtain reproducibility. Results and graphs were produced in Origin (OriginLab Corporation) and figures and tables were generated in Adobe Illustrator 2022 (Adobe).

F. Visualization and calculation of the protonation state of aerolysin

To calculate the protonation states of the aerolysin protein, we used the APBS web server[7], where we prepared aerolysin in the heptamer oligomeric state (with PDB ID: 5JZT[8]) at different pH (by using PROPKA to assign the different protonation states at pH 4, 5, 6, 7, 8 and 9) by using the AMBER force field. Once the calculation of the protein was performed, the pqr files were loaded into PyMOL[9] and the APBS Electrostatics[7] plugin (in mg-auto configuration) was used to generate the electrostatic map for the aerolysin at different pH. The pictures were generated in PyMOL[9] and UCSF ChimeraX[10], and the figure was created in Adobe Illustrator 2022 (Adobe).

Table S1

Number of charged residues on the cap domain of aerolysin.

Amino acid	Number of amino acids present in the cap	pKa of amino acid sidechain[11]
Aspartic acid (Asp)	28	4.0
Glutamic acid (Glu)	12	4.4
Histidine (His)	6	6.8
Arginine (Arg)	17	13.5
Lysine (Lys)	16	10.4

Table S2

Number of single-channel experiments performed for each pore mutants.

Pore Type	K_D	# pores observed	# Experiments
**WT pH 7.4**	2,00 x 10^{-14}	28	6
**WT pH 5**	6,20 x 10^{-14}	15	6
**K238N**	4,60 x 10^{-14}	10	6
**R220A**	2,30 x 10^{-12}	5	6
**K246C-E288C**	4,10 x 10^{-13}	0	13
**Y221G**	N/A	0	20

References

1. Iacovache, I. *et al.* Dual Chaperone Role of the C-Terminal Propeptide in Folding and Oligomerization of the Pore-Forming Toxin Aerolysin. *PLOS Pathog.* **7**, e1002135 (2011).
2. Cao, C. *et al.* Single-molecule sensing of peptides and nucleic acids by engineered aerolysin nanopores. *Nat. Commun.* **10**, 4918 (2019).
3. Smolentsev, N., Lütgebaucks, C., Okur, H. I., de Beer, A. G. F. & Roke, S. Intermolecular Headgroup Interaction and Hydration as Driving Forces for Lipid Transmembrane Asymmetry. *J. Am. Chem. Soc.* **138**, 4053–4060 (2016).
4. Lütgebaucks, C., Gonella, G. & Roke, S. Optical label-free and model-free probe of the surface potential of nanoscale and microscopic objects in aqueous solution. *Phys. Rev. B* **94**, 195410 (2016).

5. Gomopoulos, N., Lütgebaucks, C., Sun, Q., Macias-Romero, C. & Roke, S. Label-free second harmonic and hyper Rayleigh scattering with high efficiency. *Opt. Express* **21**, 815 (2013).
6. Schönfeldová, T. *et al.* Ultrasensitive Label-Free Detection of Protein–Membrane Interaction Exemplified by Toxin-Liposome Insertion. *J. Phys. Chem. Lett.* **13**, 3197–3201 (2022).
7. Jurrus, E. *et al.* Improvements to the APBS biomolecular solvation software suite. *Protein Sci.* **27**, 112–128 (2018).
8. Iacovache, I. *et al.* Cryo-EM structure of aerolysin variants reveals a novel protein fold and the pore-formation process. *Nat Commun* **7**, (2016).
9. The PyMOL Molecular Graphics System, Version 2.9 Schrödinger, LLC.
10. Pettersen, E. F. *et al.* UCSF ChimeraX: Structure visualization for researchers, educators, and developers. *Protein Sci.* **30**, 70–82 (2021).
11. Hass, M. A. S. & Mulder, F. A. A. Contemporary NMR Studies of Protein Electrostatics. *Annu. Rev. Biophys.* **44**, 53–75 (2015).
